# A perspective on multi-target drug discovery and design for complex diseases

**DOI:** 10.1186/s40169-017-0181-2

**Published:** 2018-01-17

**Authors:** Rona R. Ramsay, Marija R. Popovic-Nikolic, Katarina Nikolic, Elisa Uliassi, Maria Laura Bolognesi

**Affiliations:** 1 Biomedical Sciences Research Complex, University of St Andrews, North Haugh, St Andrews, KY16 9ST UK; 2 Department of Pharmaceutical Chemistry, Faculty of Pharmacy, University of Belgrade, Vojvode Stepe 450, 11000 Belgrade, Serbia; 3 Department of Pharmacy and Biotechnology, Alma Mater Studiorum-Bologna University, Via Belmeloro 6, 40126 Bologna, Italy

**Keywords:** Multi-target drugs, Neurodegeneration, Cancer, Cheminformatics, Virtual screening, Biological assays

## Abstract

Diseases of infection, of neurodegeneration (such as Alzheimer’s and Parkinson’s diseases), and of malignancy (cancers) have complex and varied causative factors. Modern drug discovery has the power to identify potential modulators for multiple targets from millions of compounds. Computational approaches allow the determination of the association of each compound with its target before chemical synthesis and biological testing is done. These approaches depend on the prior identification of clinically and biologically validated targets. This Perspective will focus on the molecular and computational approaches that underpin drug design by medicinal chemists to promote understanding and collaboration with clinical scientists.

## Introduction

Drug discovery in the 21st century has the disadvantage that it is very difficult to get new compounds into the clinic. The diseases where cures or at least treatments are sought are complex ones involving many potential defects in the structure, function, or regulation of the cells involved. Major targets that will be considered here are Alzheimer’s (AD) and Parkinson’s (PD) diseases of neurodegeneration, and cancer. Despite the complexity of causative factors that can lead to these disease states, the tools of modern drug discovery have the power to cover millions of compounds or fragments and determine their potential association with a target before specific chemical synthesis and biological testing is done. This perspective will focus on the molecular and computational approaches that underpin drug design by medicinal chemists. These approaches require the prior identification of clinically and biologically validated targets, and subsequent experimental testing both in vitro and in vivo. The success of drug design for complex diseases depends on an interdisciplinary and collaborative approach, and on scientists and clinicians who are willing to communicate and work together throughout the process.

## Designing multi-target drugs for complex diseases

### The transition from the single-target to the multi-target concept for drug design

Traditionally drugs have been designed with the aim of targeting a single biological entity, usually a protein (the so-called “on-target”), with high selectivity to avoid any unwanted effects arising from mis-targeting other biological targets (“off-targets”). On this basis, the concept of drugs interacting with multiple targets has long been flagged as undesirable, as it was inherently associated with adverse side effects. However, the complexity of the current incurable pathologies has clearly demonstrated that such single-target drugs are inadequate to achieve a therapeutic effect [1, 2]. In parallel, we have learned that molecules hitting more than one target may possess in principle a safer profile compared to single-targeted ones [1, 2].

Building on such accumulating evidence, the concept of multi-target drugs has made rapid and spectacular progress from being an emerging paradigm when first enunciated at the beginning of 2000 [3–5], to one of the hottest topics in drug discovery in 2017. Indeed, in the years, these concepts have triggered the interest of the drug discovery community both in academia and pharmaceutical companies to such a point that a plethora of multi-target drugs are already available on the market.

### The analysis of FDA-approved new molecular entities (NMEs) from 2015 to 2017

**Fig. 1:**
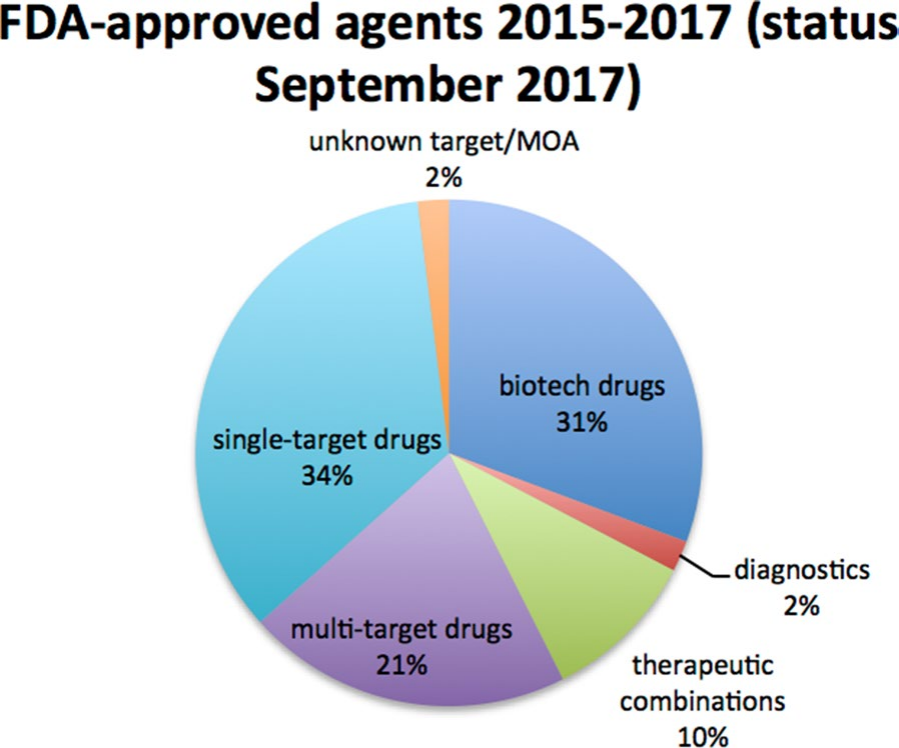
Distribution of the new molecular entities (NMEs) approved from 2015 to 2017 (status September 2017), organized according to the different classes of NMEs

As a clear proof of such translational success of multi-target drugs into the clinic, we have performed an analysis of the US Food and Drug Administration (FDA)-approved new molecular entities (NMEs) from 2015 to 2017 (status September 2017), following a similar analysis (from 2000 to 2015) made by Lin et al. [6]. The 101 new NMEs on FDA.gov approved over this triennium were classified into NME categories (small molecules, biologics, therapeutic combinations and diagnostics). By using the DrugBank database, which compiles information on approved drugs, together with their target(s) and mechanism(s) of action (MoA), the small molecules have been further analyzed and subdivided into single-target and multi-target drugs. As depicted in Fig. 1, biotech drugs (proteins, peptides and monoclonal antibodies) represent 31% of the novel NMEs, nearly approaching the number of single-target drugs (34%). This clearly reflects the recent increased pharma interest in discovery of biologics [7], which build on the premise of a personalized treatment [8].

**Fig. 2:**
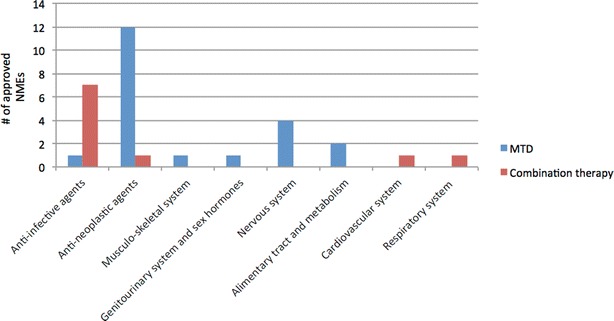
The 21 multi-target drugs and 10 therapeutic combinations approved in 2015–2017 organized according to their Anatomical Therapeutic Chemical (ATC) Classification System

Notwithstanding the increase of approved biologics in recent years, small molecules, both single-targeted and multi-targeted ones, continue to contribute. Although the number of single-target small molecules (34%) is still greater than that of multi-target drugs (21%), the latter keeps increasing compared to previous years (16%) [6, 9]. However, if we broaden our view and reason in terms of general polypharmacology, we can add together the 21% of multi-target drugs and the newly approved therapeutic combinations (10%). In this way, the total percentage (31%) approaches that of single-target drugs (34%). This unequivocally supports the attractiveness of polypharmacological strategies, especially in certain therapeutic areas, such as anti-infective, nervous system, and anti-neoplastic agents (Fig. 2).

### Examples of multi-target drugs for complex diseases

With regards to anti-infectives, daclatasvir is one of the last approved (2015) non-interferon based agents against hepatitis C virus (HCV), developed to selectively target one of the key viral proteins involved in the HCV replication, i.e. nonstructural 5A (NS5A) phosphoprotein [10]. Despite the high effectiveness, several combination therapies have been approved since January 2016 because drug-resistance is an already established phenomenon [10]. These combinations were all conceived based on the prototypical polypharmacology hypothesis that targeting two different nodes of HCV replication may exhibit superior antiviral activity compared to single drugs and reduced resistance [11]. These combinations include: (i) elbasvir, a NS5A inhibitor, combined with grazoprevir, a NS3/4A protease inhibitor, (ii) the combination of sofosbuvir, a nucleotide analogue NS5B polymerase inhibitor, and velpatasvir, a NS5A inhibitor, and (iii) the fixed-dose combination of glecaprevir, an NS3/4A protease inhibitor, and pibrentasvir, an NS5A inhibitor [11].

Nervous system application of polypharmacology has led to approval of four multi-target drugs. Recently, an increased number of multi-target drugs have been developed to treat schizophrenia and major depressive disorders [12–14]. The main strategy for treatment of schizophrenia is based on antagonizing dopamine D_2_, serotonin 5-HT_2A_ and α1-adrenergic receptors [15]. Incorporating effects at these broad targets has the objective of either improving antipsychotic efficacy or mitigating adverse effects. With these concepts in mind, starting from the arylpiperazine substructure as suitable scaffold for achieving a fine balancing of D_2_, 5-HT_1A_ and 5-HT_2A_ receptors activities, the prototype of the third-generation of antipsychotics, aripiprazole, launched into the market in 2015, was the first designed serotonin-dopamine activity modulator (SDAM). However, it also displayed unwanted side effects, probably arising from a sustained interaction with post-synaptic D_2_ receptors. Thus, brexpiprazole was licensed on October 2015 as a novel D_2_ and 5-HT_1A_ partial agonist, with less intrinsic activity at D_2_ receptors and more balanced activities at 5-HT_2A_, 5-HT_1A_, and α_1B_ receptor subtypes than aripiprazole [15].

Another excellent example is cariprazine, approved by the FDA in September 2015 for the treatment of schizophrenia and bipolar disorders. Similarly to the above mentioned SDAM, cariprazine shares the arylpiperazine substructure linked to an alkyl-*N*′,*N*′-dimethyl urea, which determines a profile of partial agonism at D_2_ and D_3_ receptors [16]. This mechanism is innovative because many other antipsychotics are only D_2_ and 5-HT_2A_ partial agonists (see above).

Concerning neurodegenerative disorders, the polypharmacological approach looks particularly promising, given the complexity and multifactorial nature of such diseases and their unknown etiopathogenesis [17, 18]. For example, rasagiline, approved in 2006 for neurodegenerative diseases, displayed a multi-target profile. The first new chemical entity approved for neurodegeneration in over a decade is a multi-target drug called safinamide. Safinamide was originally developed as anticonvulsant agent [19] and approved in March 2017 as an adjunctive treatment for PD thanks to its particular multi-target profile. It combines dopaminergic effects, including selective and reversible MAO-B and dopamine reuptake inhibition, which are largely responsible for its effects on motor symptoms, along with non-dopaminergic properties, i.e. blockade of voltage-dependent Na^+^ and Ca^2+^ channels and consequent inhibition of glutamate release that is thought to confer neurorescue and neuroprotectant effects [20].

Apart from schizophrenia and neurodegeneration, another key disease that would benefit from polypharmacological strategies is cancer, where several aberrant proteins and pathways concur to initiate tumor growth and to facilitate its progression. Similarly to the above mentioned diseases, redundancies and complexities of biological pathways often lead to compensation and resistance to targeted therapies [21–23]. Among the different classes, multi-kinase inhibitors emerged as the most exploited anti-cancer polypharmacological approach, followed by pan-inhibitors of histone deacetylases (HDACs). Lenvatinib in the first group is a reversible multi-tyrosine kinase receptors inhibitor that modulates the activities of vascular endothelial growth factor receptors (VEGFR) 1–3, fibroblast growth factor receptors (FGFR) 1–3, RET, mast/stem cell growth factor receptor kit (SCFR), and platelet-derived growth factor receptor (PDGFR) beta, all implicated in pathogenic angiogenesis, tumor growth, and cancer progression [24]. Given the broad activity profile, it was approved for the treatment of radioiodine-refractory thyroid cancers. Neratinib is another recently approved multi-tyrosine kinase inhibitor with an irreversible mechanism of action, which exhibits antitumor activity by targeting epidermal growth factor receptor (EGFR), and human epidermal growth factor receptor 2 (HER2), both highly expressed in several carcinomas. Taking advantage of the high sequence identity shared by EGFR and HER-2 (82%) in the ATP domain, the design of such dual-inhibitor bearing a Michael acceptor warhead was undertaken. Computational studies guided the optimization of this molecule so that its warhead is positioned suitably to interact with Cys 773 of EGFR and the analogous Cys 805 of HER-2 [25].

Another well-explored class of multi-kinase inhibitors is represented by dual inhibitors of cyclin-dependent kinase (CDK) 4 and 6. Starting from the pyrido[2,3-d]pyrimidin-7-one scaffold as template for the inhibition of a broad cross-section of kinases, including pan-CDKs inhibitors which have been discontinued due to the associated toxicity, through a combination of chemical screening and optimization, it was found that the modification at the C2-position provides inhibitors with exquisite selectivity for CDK 4/6. Among them, palbociclib, abemaciclib and ribociclib were approved by FDA as breast cancer therapy in this time frame [26].

Midostaurin, a semi-synthetic derivative of the pan-kinase inhibitor staurosporine, is a well-known multi-kinases inhibitor, approved on April 2017 for the treatment of those adult patients with newly diagnosed acute myeloid leukemia that have a specific variant of the FLT3 gene. It was shown to inhibit the activity of protein kinase C alpha (PKC alpha), VEGFR2, KIT, PDGFR and WT and/or mutant FLT3 tyrosine kinases [27].

Regarding the hot topic of epigenetic polypharmacology [28], panobinostat is a cinnamic hydroxamate-based histone deacetylase inhibitor (pan-HDAC inhibitor) approved on February 2015 by the FDA for the treatment of multiple myeloma in combination with bortezomib (a proteasome inhibitor) and dexamethasone. Nonselective inhibition of both classes (I and II) of HDAC enzymes resulted in increased acetylation of histone proteins, leading to cell cycle arrest and/or apoptosis of cancer cells. However, it is interesting to note that its broad activity profile against HDACs, which depends on zinc chelation for activity, lacks therapeutic efficacy as monotherapy in patients with multiple myeloma, whereas synergistic activity has been demonstrated when used in combination with other drugs targeting different hubs of the tumor network [29]. This lends support to the concept that the network disruption approach is highly valuable in anti-cancer drug discovery.

### Current challenges of rational design of multi-target drugs

From these data, we can conclude that the time is ripe to extend applicability of multi-target drugs and polypharmacology across different therapeutic areas. However, how to develop them in a rational way is still a big challenge, both in terms of target selection and small molecule discovery [30–32]. Regarding the first issue, although there is a variety of valuable online resources [33–35], it is still a challenge to choose the right combination of targets for the disease of interest in both multi-target drugs and therapeutic combinations. It requires good understanding of target-disease associations, pathway-target-drug-disease relationships and adverse events profiling [36]. Furthermore, the selection should be based on whether or not modulating the selected targets could lead to additive or synergistic effects [37]. Particularly, additive effects can be obtained when the targets belong to the same pathway, whereas synergism can be achieved only if the selected targets are located on functionally complementary pathways. This means that in both cases the effect is attained at lower doses, and consequently a better safety profile can be expected with respect to single-target drugs [37].

**Fig. 3:**
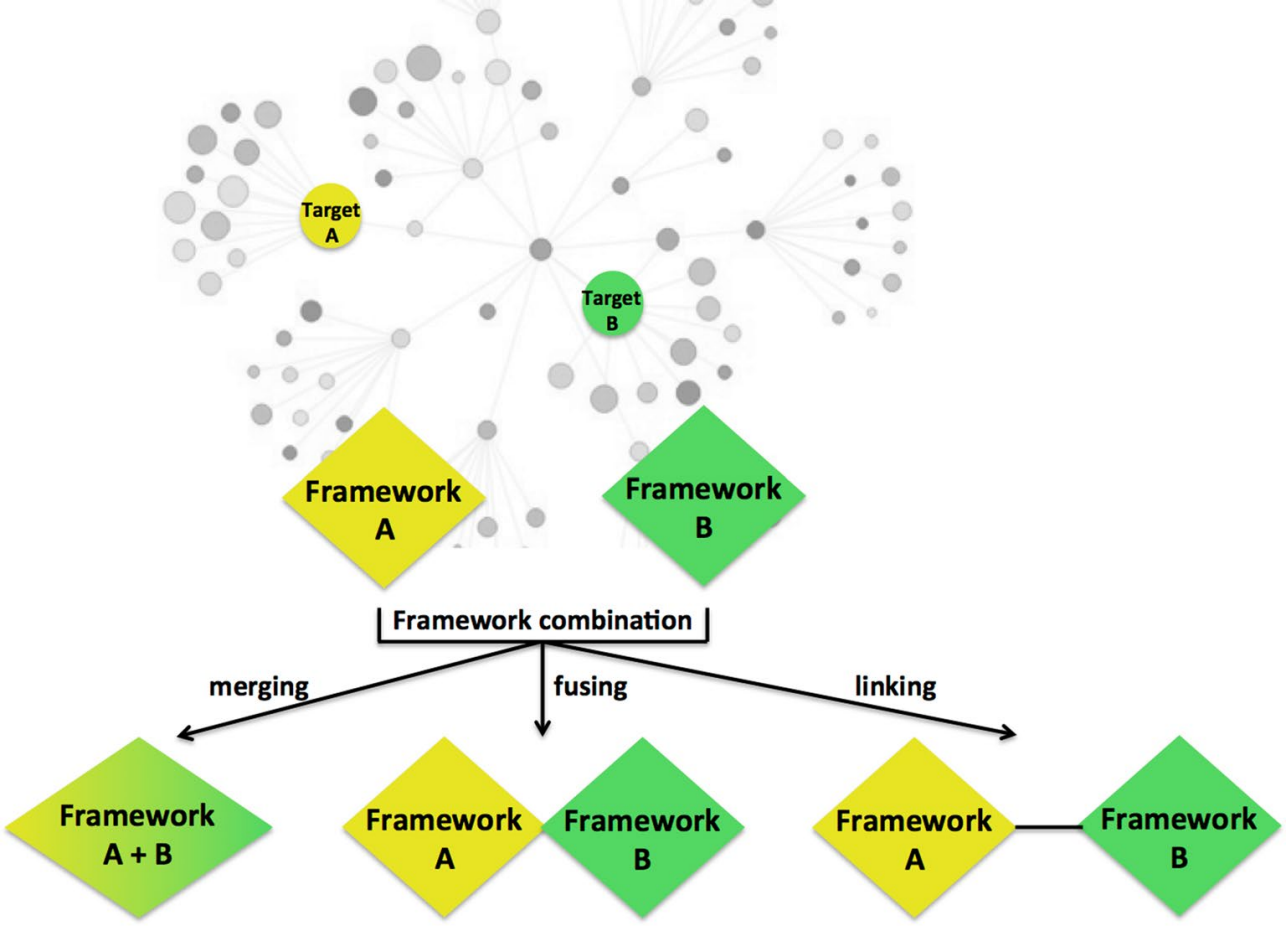
Rational design of multi-target compounds

With regards to the second issue, although early drugs were discovered serendipitously, multi-target compounds are now rationally designed, typically by combining two distinct frameworks into a single chemical entity [38–40], starting from compounds with the desired activity towards the targets of interest. Thus, multi-target compounds routinely result from the integration of pharmacophores of selective molecules, either already known drugs or drug candidates (Fig. 3). Intuitively, pharmacophores featuring similar scaffolds, usually ring systems, can be fused or merged depending on the overlapping degree between the starting frameworks. Alternatively, if pharmacophores present different structural elements required for the interaction with each target, they can be conjugated with cleavable or non-cleavable linkers, although this strategy often leads to molecules with poor drug-likeness properties [41]. In all cases, the generation of multi-target compounds is typically driven by the nature of the targets, the availability of starting frameworks and the chemical tractability. Beyond that, the essential requirement of the multi-target compounds is that each framework retains the ability to interact with its specific target [32]. This undoubtedly challenging task involves considering structure–activity relationships that govern the interaction of the starting molecules with their specific targets, and becomes most challenging when these targets are only slightly related or unrelated (i.e. when they belong to different protein families) [32].

Furthermore, establishing the same degree of modulation for each target (i.e. balancing the activities towards the targets) and “designing out” unwanted interactions to any undesired target (off-target) are critical factors to be addressed. The latter aspect is of particular importance when designing multi-target compounds directed to targets belonging to the same family (e.g., multi-kinases inhibitors), but also in the case of shared functional domains and/or binding sites across target families [32]. Clearly, the rational design of multi-target compounds is far from being an easy task, dealing with the crucial issues of selecting the right target combination, achieving a balanced activity towards them, and excluding activity at the undesired target(s), while at the same time retaining drug-like properties.

## The tools to identify hits/leads

### Computational approach to ligand discovery and back validation of hits by docking

Rational drug design is extensively applied in the search for novel agents as an efficient tool for hit identification, validation, optimization, and evaluation. Computational approaches such as cheminformatics and virtual screening, pharmacophore development, molecular docking and molecular dynamics are mainly used to identify new agents, define molecular determinants for enhanced activities, design and evaluate more efficient drugs with improved safety [42].

The structure-based in silico methods use structures of ligand-target complexes to identify the structural origins of activity and selectivity of related ligands. Ligand-based computational methods are able to determine essential structural features for a set of bioactive ligands and identify the structure of potential lead molecules [43]. Novel in silico methods that combine ligand and structure activity relationship methods provide the most comprehensive information about drug-target interaction and significantly increase the success rate of the rational drug design [44–46]. Different computational approaches for ligand discovery have been developed helping to reduce late stage attrition and to limit the number of expensive and time consuming experiments required to synthesize the active novel hits with optimized pharmacodynamic and pharmacokinetic properties [47]. The importance of wide application of computational methods in drug discovery processes has been extensively reviewed [48, 49].

A commonly used cheminformatics methodology for lead identification is similarity-based. Molecular similarity between chemical structures from in silico libraries and molecules already known to possess activity against protein target is used to predict bioactivity. Unlike the simplest approach for single-targets, this method can be modified for application on multi-target problems with some missing data. In that case, the researchers do not base their search on single known active compound, but rather on families of compounds [50]. Another modification that can be applied to multi-target problems is a quantitative method of estimating the probability that a certain molecule is associated with the particular bioactivity-scaffold combination defining one specific refined family [49]. Subsequently, more complex methods can help to make comparable predictions of not only a query activity but also molecules with off-target and promiscuous interactions. This approach was successfully implemented for the identification of performance-enhancing molecules that should be prohibited in sports [50], and for predicting multi-target bioactivities of potential polypharmacological compounds for treatment of neurological diseases [51]. Cheminformatics-based target identification has been applied for identification of multi-target ligands intended to interact with MAO-A and MAO-B; acetylcholinesterase (AChE) and butyrylcholinesterase (BuChE); or with histamine *N*-methyltransferase (HMT) and histamine H_3_-receptor (H_3_R) [51].

### Quantitative structure–activity relationship (QSAR)

**Fig. 4:**
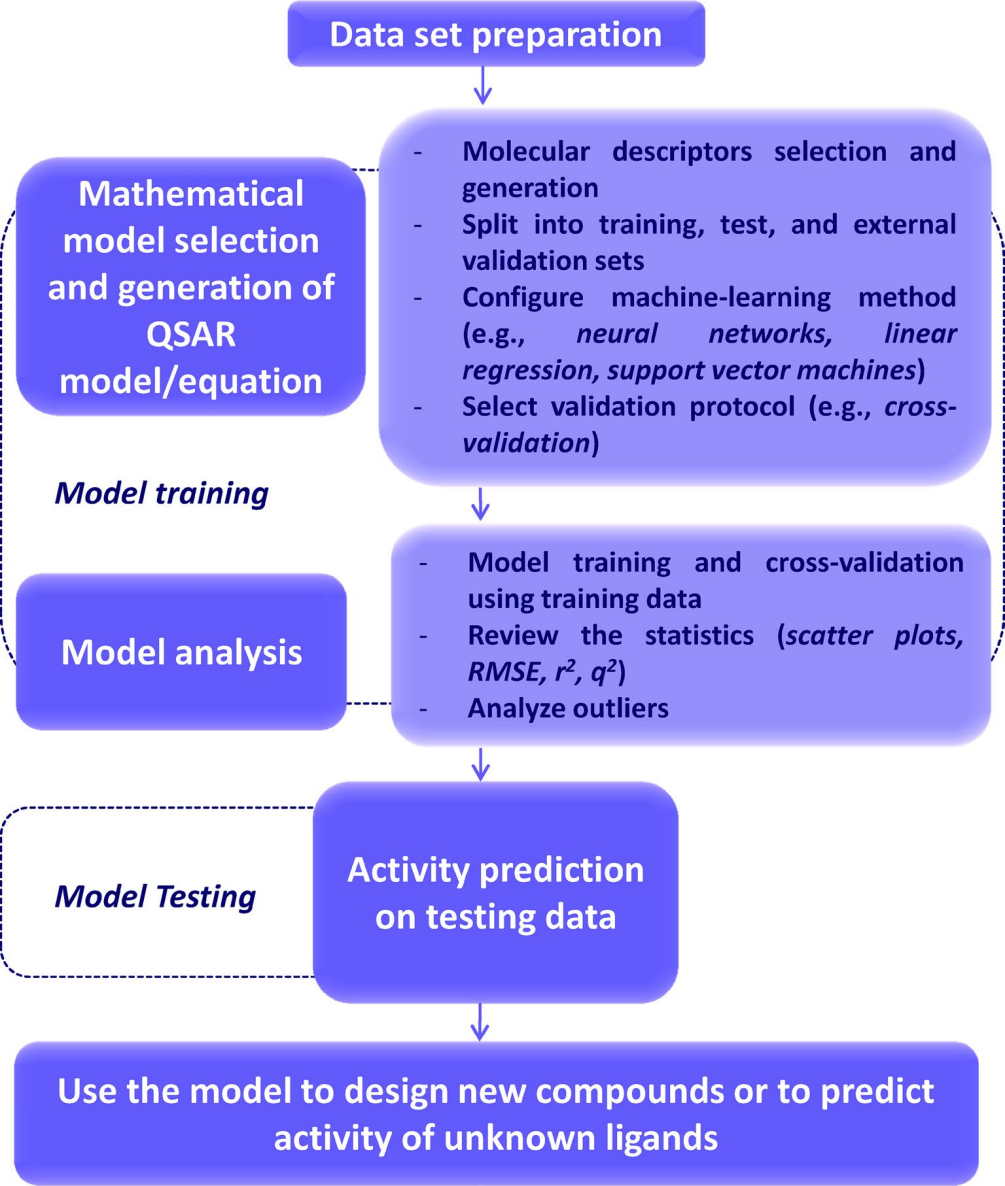
Schematic representation of the QSAR workflow

Quantitative structure–activity relationship (QSAR) modelling, widely used for development of the biological and physical properties of new compounds, is a crucial initial step in lead optimization to correlate molecular structure with biological and pharmaceutical activities (Fig. 4). This approach selects molecular descriptors that are representative of the molecular features responsible for the relevant molecular activity. The usefulness of these descriptors in QSAR studies has been extensively demonstrated, and they have also been used as a measure of structural similarity or diversity [45, 47]. The 2D-QSAR methods require lower calculation times which allow them to be used mostly as preliminary filters to reduce the number of compounds that require further screening in later stages of drug development. The 3D-QSAR approach is used to construct a tridimensional structure of the pharmacophore and to define the functional links between the 3D-molecular determinants and the activity [47]. The important advantage of QSAR modeling is an understanding of the effect of chemical structure on activity. When a large amount of experimental data is available, QSAR makes possible the selection of the best candidates for the synthesis of novel compounds. Interpolation can be applied, but it must be taken into account that extrapolation should not be extended outside the scope of the data set. The results obtained in this way can be used to understand the productive interactions between functional groups in the molecules and those in their target. On the other hand, QSAR modeling can have several disadvantages. False correlations can occur if the biological data used include experimental errors. Sometimes QSAR results cannot select the most active compounds with a suitable degree of confidence if the underlying experiments do not include a complete sense of experimental design [41, 42, 47]. Successful examples of the 3D-QSAR approach and related chemoinformatic methods application are described in comprehensive overview about the development of tacrine- and donepezil-like multitarget compounds for the treatment of AD [44, 52].

### Virtual screening (VS)

Virtual screening (VS) is a reliable, inexpensive method for identifying leads by providing the screening of whole libraries of small molecules in order to get practically achievable number of compounds with the structures of the highest probability of binding to a drug target [53]. VS, a computational method with an essential role in drug discovery and an alternative to experimental high throughput screening, can be classified into two techniques: ligand-based virtual screening (LB-VS) and structure-based virtual screening (SB-VS). LB-VS approaches are based on analysis of a miscellaneous set of ligands in order to define pharmacophore for further score and are usually applied when no structural information about the target protein is available. SB-VS can be employed when the three-dimensional structure of target protein is known from X-ray crystallography or nuclear magnetic resonance (NMR) spectroscopy. This technique involves molecular docking of each ligand into the binding site, followed by ranking of the compounds based on their likely affinity for the biomolecular target. The techniques of VS often require construction of an in silico library of compounds which could readily be synthesized once they have been selected from a virtual screen [54].

Examples of application of SB-VS in lead discovery have been reviewed by Sliwoski et al. [53]. One of the most successful is SB-VS of 0.7 million compounds from the database rCat [55] with Hsp90, an important therapeutic target for oncology, which allowed identification of lead compounds and their development to potent inhibitors of Hsp90. In another example, a homology model of M1 acetylcholine receptor (mAChR) based on crystal structure of bovine rhodopsin, served for VS and led to development of a series of novel 1-(*N*-substituted piperidin-4-yl) benzimidazolones [53].

In addition to independent application, the combination of the structure- and ligand-based VS strategies can be also implemented in lead identification studies where all available chemical and biological information are taken into account. Lepailleur and co-workers applied pharmacophore-based virtual screening in combination with similarity based clustering method and molecular docking to identify dual H_3_R antagonist/5-HT_4_R agonists [56]. Binding experiments confirmed that benzo[h]-[1,6]naphthyridine ligands selected by this VS approach exert high affinity for both H_3_ and 5-HT_4_ receptors. Recently, Bottegoni et al. [57] carried out a VS protocol to identify fragments that display considerable activity at both β-secretase1 (BACE-1) and glycogen synthase kinase 3β (GSK-3β), two structurally unrelated enzymes associated with the onset of AD.

Perhaps one of the best examples that describe the application of computer approaches in drug development is ligand discovery for the treatment of AD. A virtual ligand screen was applied for lead optimization of two donepezil hybrid compounds that inhibit MAO-A, MAO-B, AChE, and BuChE. The experimental results confirmed the quality of the 3D-pharmacophores showing that the most potent against all four enzymes were the compounds selected by the models from previous 3D-QSAR analyses as the best candidates [58, 59]. The synthesis and pharmacological evaluation of new donepezil-indolyl hybrids [58, 59] and donepezil-pyridyl [60] as multifunctional drugs for the potential treatment of AD, using the multipotent reference compound ASS234 have also been reported by the same group. Potential for development of multiple ligands for dopamine receptors involved in numerous neurological disorders was comprehensively reported by Butini et al. [61]. Rational design and a computational approach were used for identification of a new series of 1,2,4-triazol-yl-azabicyclo[3.1.0]hexanes as a D_3_ receptor antagonists [62] with high in vitro affinity and selectivity at the D_3_ receptors. On the basis of the profile of aripiprazole a series of new antipsychotics with affinities at D_3_ and D_2_ receptors were developed [63], where compound cariprazine has passed clinical evaluation and was approved by the FDA for schizophrenia and bipolar disorders in 2015.

The most important advantages of VS compared to laboratory experiments or high-throughput screening (HTS) are capacity for testing large in silico libraries of compounds, price, speed, and safety. The VS analysis can be applied to reduce the initial number of compounds before using HTS methods. VS approaches allow investigation of compounds that have not yet been synthesized or purchased. Some disadvantages of VS arise from the fact that VS cannot completely replace the experimental HTS which can test the activity of hundreds to thousands of compounds against the target and which provides essential results for further drug discovery.

### Molecular docking

To confirm all predictions and perform back validation of hits, molecular docking studies can be conducted before compound synthesis. Molecular docking is a computational technique that can be used to model the interaction between a small molecule and a target protein at the atomic level, subsequently providing the prediction of which conformation best fits the protein binding site and an estimate of the stability of the ligand–protein complexes [64]. The most often used docking software packages are CDOCKER [65], GOLD [66] and AutoDock [67]. Knowing the location of the binding site beforehand allows a small part of the protein to be studied and significantly increases the docking efficiency. Generally, objective functions are calculated and used to analyse possible conformations of ligands, to search for the most stable binding modes of the ligands with the target, and to optimize the geometry of docked ligand-target complexes. It would be too expensive to generate all the possible conformations, so various sampling algorithms are developed to reproduce the binding mode and to provide scoring functions to rank all generated conformations. Programs based on different algorithms have made docking an increasingly important tool in pharmaceutical research.

The main advantages of using molecular docking methodology are to analyse the binding modes of the ligand with the target, compare binding energies of various ligands, and estimate stability of docked ligand-target complexes. The objective and scoring functions used in docking reflect the fact that binding of a ligand to its target is partly based on their chemical complementarities and physicochemical interactions, and partly on shape complementarities, which may well be conformation-dependent. Flexibility of the target biomolecule can be modelled either by performing virtual docking to set of rigid protein conformations or by examining dynamic ligand-target complexes. A combination of molecular docking and molecular dynamic studies can therefore be used to select the key interactions between the ligands and the targets that must be retained in new molecules.

Examples of successful implementation of the docking simulations can be illustrated by the studies of novel multi-target compounds for neurodegenerative diseases (Table 1). Molecular modeling analysis and docking simulations, using the program Autodock Vina and the Protein Data Bank crystal structures of four enzymes (AChE/BuChE/MAO-A/MAO-B), conducted on a series of experimentally synthesized donepezil-indolyl hybrids [59] and donepezil-pyridyl hybrids [60] revealed that compounds DIH15 [59] and DPH14 [60] expressed the best observed drug-like characteristics and might be considered as a promising compounds for further development for the treatment of AD. Moreover, a series of docking simulations identified the most promising donepezil hybrid [68] as an interesting lead compound for the design of novel MTDL for AD therapy, ready for experimental validation. The subsequent experimental validation of predicted hits is the only indicator of actual reliability of implemented computational approach and represents the critical step which should be a standard component that follows any ligand discovery process [45].

**Table 1:** The examples of successful docking studies used in ligand discovery for the treatment of AD

Novel ligand	Target	PDB	Ligand-receptor interactions	Refs.
Donepezil-inolyl hybrid (DIH15)	AChE	1C2B	Tyr124, Tyr341, Phe338, Trp286, Tyr72	[59]
	BuChe	2PM8	Ser198, Hys438, His438, Trp82, Trp231	
	hMAO-A	2Z5X	Glu215, Val93, Leu97, Ala111, Tyr407, Tyr444, FAD, Cys323	
	hMAO-B	2V5Z	FAD, Leu 88, Pro102, Ile316	
Donepezil-pyridyl hybrid (DPH14)	AChE	1C2B	Trp286, Tyr124, Trp86, Tyr124, Tyr337, Tyr341, Tyr72, Tyr124, Asp74	[60]
	BuChe	2PM8	Trp82, Ser198, Leu286, His438, Tyr332	
	hMAO-A	2Z5X	Tyr407, Tyr444, Gln215, Ser209, Val93, Leu97, Ala111	
	hMAO-B	2V5Z	Tyr398, Tyr435, Gln206, Cys172, Pro102, Thr201, Thr314, Ile316	
Donepezyl hybrid (compound 5)	AChE	1EVE, 2CKM, 1Q83	Trp279, Trp84, Tyr334, Asp72, Tyr70, Phe330	[68]
	BuChe	2PM8	Trp79, Phe330, Tyr70, Phe290, Trp279	
	hMAO-A	2Z5X	Phe208, Ile325, Leu97, Leu337, Val210, Cys323, Arg109, Gly110	
	hMAO-B	2BYB	Ile199, Ile316, Tyr326, Arg100, Gly101, Glu84, Leu88	

### Computational approaches to druggability

Multi-potent inhibitors of cholinesterases and monoamine oxidases were proposed as novel agents for therapy of AD and PD [51, 59, 68–71]. Ladostigil and rasagiline, drugs acting as inhibitors of monoamine oxidase, are propargylamine derivatives able to retard neurodegeneration and decrease deposition of Aβ-containing plaques. Therefore, these agents were selected for clinical trials as novel drug candidates in therapy of AD. The completed phase II clinical trials for ladostigil (NCT01354691) and rasagiline (NCT00104273) were an important proof of concept for MTDL as novel drugs in therapy of AD.

The effectiveness of CNS drugs depends on drug penetration through the blood–brain barrier (BBB) between the blood capillaries and brain tissue, the extent of distribution of the drug in the brain, and the drug activities on the targets. These multiple factors increase the complexity of CNS drug discovery [72]. The main parameter of interest is the unbound brain concentration (C_u,b_) of the drug. The C_u,b_ can be directly related to the drug concentration at the target site and thus to in vivo drug efficacy. Receptor occupancy is also very important experimental descriptor for evaluating target engagement by the examined drug. Total brain concentration (C_b_) is only used as measure of the nonspecific binding of the drug to brain [73, 74]. The development of quantitative structure-exposure relationships between molecular parameters of drugs and experimental parameters of their brain exposure is now a valuable tool for predicting CNS drug efficacy [74, 75].

The most important physicochemical factors for CNS drug penetration in brain and for efficacy are optimal lipophilicity, hydrogen bonding, aqueous solubility, p*K*_a_, and molecular weight of agents. Moderate lipophilicity of CNS drugs at physiological pH [cLogP: 2–5, cLogD (pH 7.4): 2–5] facilitate transport through BBB [76], while higher lipophilicity increase plasma protein binding, decrease solubility in plasma, and rise metabolic and toxicity risks of the agents [77].

Hydrogen bonding parameters, such as hydrogen bond donor (HBD) and hydrogen bond acceptor (HBA) counts, are dominant descriptors for unbound brain concentrations (C_u,b_) of agents as crucial measure of in vivo CNS drug efficacy [78]. Reducing the HBD and HBA counts (HBD < 3; HBA < 7) [72, 76] of a drug is a key step applied in optimizing drug brain exposure [79]. Moderate lipophilicity (cLogP < 4) together with considerable topological polar surface area (TPSA 40–80 Å^2^) are defined as optimal combination of parameters for increased unbound brain concentrations of CNS drugs [80]. Aqueous solubility can also be examined in combination with the above molecular properties. Most CNS drugs showing aqueous solubility of more than 100 μM have low safety risk [81].

Current CNS drug discovery applies computational approaches to propose structural modifications of investigated compounds for better brain penetration and in vivo drug efficacy. Design of CNS drug candidates with optimal balance between physicochemical properties for efficient brain exposure is now the big challenge in CNS drug discovery [82–84]. Thus, future CNS drug design will include developments of predictive computational methods and exploring of CNS property space for more efficient penetration in brain and enhanced efficacy [74, 75].

## Biological validation of leads

The biological side of drug discovery is intimately integrated to the process of high-throughput compound assessment. Computational searches are underpinned by the prior identification of targets for specific medical conditions and by the crystal structures or at least a detailed pharmacophore derived from in vitro structure–function studies. The large numbers of hits from computation searches then require high throughput assessment using chemical libraries and assays for the specific targets. This section will focus on the types of assays used in small-scale academic laboratories, but adaptation to high throughput and multiplexed methods are now also essential for translational drug discovery.

Most disease-modifying targets can be divided into the three categories: receptors, enzymes, or macromolecule interactions. For all of these assessing binding is the primary goal. The initial hits from binding (either computationally or experimentally determined) are then further discriminated using more in depth methodologies. The biological information is fed back into the chemical development to optimize the hits to provide lead compounds to explore for toxicity and for efficacy in higher-level systems. It is at the hit-to-lead stage that academic groups both in medicinal chemistry and in biological and medical sciences can make substantial contributions, particularly with the improved biophysical technologies now available [85, 86].

The initial screening of compounds for biological activity should ideally give values that can be compared with other studies. Limitations on materials or time are often restrictive when screening large numbers of compounds. Table 2 compares some parameters that can be obtained for receptors, enzymes, proteins and cells at the simplest level. Measuring effects that result from the association of ligand and its target can be more convenient, and is often more informative, certainly for receptors, the first of the targets to be considered below.

**Table 2:** Increasing complexity of measurements for assessing new compounds in vitro

Target	Parameters	Effects
Receptor (R)	$L + R\mathop{\rightleftarrows}\limits_{k_{\text{off}}}^{k_{\text{on}}}LR \to {\text{conformational change}}$	Multiple effects possible: on, off, partial, etc
Enzyme (E)	Reversible $L + E\mathop{\rightleftarrows}\limits_{k_{\text{off}}}^{k_{\text{on}}}{\text{LE}}$ Irreversible $L + E \mathop{\rightleftarrows}\limits_{k_{\text{off}}}^{k_{\text{on}}}{\text{LE}}\xrightarrow{{k_{\text{inact}} }}L - E$	Decreases product, prevents depletion of substrate but can be out-competed by substrate Inactivated enzyme; recovery depends on normal turnover of enzyme (minutes to days)
Protein or DNA (M)	$L + M\mathop{\rightleftarrows}\limits_{k_{\text{off}}}^{k_{\text{on}}}LM$	Ligand and target often at same concentration in vitro. Outcome of binding best measured downstream
Cell target (T)	$L_{\text{out}}\xrightarrow{\text{cell uptake}}\,L_{\text{in}} \xrightarrow{\text{metabolism?}}\frac{\text{Organelle uptake}}{\text{Other binding}} \to L + T\mathop{\rightleftarrows}\limits_{k_{\text{off}}}^{k_{\text{on}}}{\text{LT}}$	Concentration at target site unknown Major effects often easy to assess (e.g., cell death) but mechanism can be obscure

### Receptors

In the last 20 years, advances in cloning and purification of membrane-bound proteins and in crystallography and other methods of structural determination have enabled the structural characterisation of large numbers of receptors. The GPCR family has moved on from a few structures and use of derived homology models to a database of structures and experimental parameters, and full sets of expressed receptor subtypes [87–89]. Many small commercial labs now offer screening services for academic researchers. With one GPCR-targeted AD drug in use (memantine, a NMDA receptor agonist), GPCR ligands are also interesting for multi-target compounds. A recent example for AD added a histamine receptor antagonist moiety to cholinesterase and monoamine oxidase inhibition [90]. However, it is the downstream effects and integration of signalling that are important. For a given cell, the challenge of understanding the action of multiple ligands on many GPCRs linked to multiple signaling routes can now be addressed using systems biology approaches to deconvolute the spatiotemporal phenomena of intracellular signal integration [91].

Designing ligands for GPCRs is not a trivial task. Understanding exactly how ligands induce agonism or antagonism involves complex allosteric effects [92]. Progress in this area is currently an exciting area of drug design (for example, [93]) producing more specific ligands for pharmacological use. The flexibility of the GPCR proteins also leads to in vivo complexity. Transient dimerization [94], heterodimers [95], and internalized megamers [96] are now beginning to be explored both to understand the signaling involved in cells and in vivo, and to design selective ligands that promote different aggregation states.

### Enzymes

Since almost half of current drugs work on enzymes, evaluation of binding to active sites and the effect on the kinetics of the enzyme remain primary in vitro tools. Evaluation of binding to purified enzymes by surface plasmon resonance (measuring on- and off-rates and affinity constants) is the major high through-put tool but isothermal titration calorimetry (ITC, measuring binding affinity and stoichiometry) is also used. As for receptors, tuning the functional effect of binding is even more important. Ligands that compete with the native substrate are relatively easy to design but effective drugs are usually non-competitive or have a long residence time (slow off-rate) or are irreversible inhibitors of enzyme activity [97–99]. Assessment of the enzyme kinetics usually uses a specific assay detecting the change in the substrate or (better) in the product using absorbance or fluorescent detection. Increased sensitivity for an assay can be devised by designing fluorescence probes or by coupling the assay product to another (excess) enzyme that converts the product into something measurable. The desired output in initial compound screening is an IC_50_ value. This is a simple way of comparing a series of compounds assayed under the same conditions but does not allow comparisons with other series or work using other assays. For enzymes, it is much more informative to measure defined parameters (such as K_D_ or K_i_) that can be compared with computed binding constants, and can be used in modelling complex pathways. It should be noted that the relationship of IC_50_ to K_i_ from enzyme kinetics depends on the mechanism of inhibition [100].

### Cell-based assays

Designing drugs to combat neurodegeneration has always looked to cell survival as an ultimate target because cell death is involved both in PD (specifically in the substantia nigra) and in AD (general neuronal loss). Anti-oxidant activity, a target of early research, is still seen as desirable in compounds such as rasagiline. Further, the propargyl moiety of l-deprenyl was found to have neuroprotective effects, up-regulating cell survival pathways [101, 102].

Testing effects of chemicals on mitochondrial health requires more sophisticated methods. The primary goal is to protect ATP generation, best measured in intact cells by the respiratory capacity and the response to probes of mitochondrial oxidative phosphorylation. Using neuronal or glial cell cultures, the parameters of ATP turnover, proton leak, and maximum respiration rate can be determined using a dedicated analyzer (Seahorse XF) to measure the oxygen consumption rate (cell respiration) and extracellular acidification rate (an indicator of glycolysis) and how these respond to additions of oligomycin and respiratory inhibitors [103]. Live cell imaging with a chemical probe can be used to determine reactive oxygen species (ROS) production (a sign of damaged mitochondria or inhibited electron transport) and mitochondrial membrane potential. It is particularly valuable for visualizing mitochondrial movement [104] or morphology (MitoRed staining) that is an indicator of mitochondrial health or stress. Other biochemical methods augment the information, such as mRNA or protein levels of proteins involved in mitochondrial movement, biogenesis, fission and fusion (for example, [105, 106]). When the target is inside mitochondria, designing compounds to deliver a drug specifically to mitochondria is possible. Compounds with a shielded positive charge will penetrate the membranes but be accumulated in response to the membrane potential as shown for the neurotoxin MPP^+^. This strategy was exploited in the design of a compound to treat malignant gliomas [107].

The uncertainty for testing in complex systems is larger than for isolated or over-expressed enzymes or receptors, so in practice, IC_50_ values or the concentrations required to give a fixed endpoint are used for comparisons within a series of compounds. With uptake, compartmentation, non-specific binding, and metabolism to contend with, defined conditions and suitable statistical tests (and sample size) are essential.

Extending cell-based assays to organs or model animals introduces even more complexity, but a study of the toxicity of anti-inflammatory drugs showed reasonable concordance of toxic/non-toxic compounds in isolated mitochondria, rat hepatocytes, and a zebrafish model [108]. Metabolism can vary by species: for example, a novel propargylamine compound with pro-cognitive properties, *N*-(furan-2-ylmethyl)-*N*-methylprop-2-yn-1-amine (FMPA), was stable when incubated with human microsomes but was rapidly metabolized by rat microsomal CYPs [109]. For practical testing of large numbers of compounds in complex systems, the organ-on-a-chip is on the horizon [105]. It will allow deeper probing of action in a system close to reality but not yet in vivo, thus reducing the need for large numbers of animal experiments, and improving the foundation for the choice of compounds for in vivo studies.

## Conclusions

There is ongoing need to find more effective drugs to treat infection where adaptation of the invader resulting in resistance is a problem, to combat cancer where advanced understanding of the mechanisms now requires selective combinations for specific cancers, and to halt progression in AD where only symptomatic therapies are available. Computational methods have enormously increased the rational approach to drug design in recent years, building on the molecular structure determinations of previous decades (a process still ongoing for membrane proteins such as receptors). QSAR methodology has been applied to define molecular determinants for ligand effects on the target, to design novel structures, and to select the best candidates for further studies. Virtual screening approaches have been used to rank the designed compounds with big databases and for lead searches in large in silico libraries of compounds. Based on the molecular docking, dynamic studies explain binding, compare stability of the ligand–protein complexes, and enable determination of interactions important in the binding modes of the ligands.”

In diseases where single-target drugs have failed or show severe limitations, multi-target drugs emerge as more effective therapeutics. Herein, we have outlined current medicinal chemistry approaches that seek the initial tools to better define causative mechanisms and targets to which novel multi-target drugs should be directed. Prediction of the correct targets to combine relies on clinical experience from single–target drugs, an area that should be boosted considerably by quantitative systems pharmacology, a computer-based methodology that combines preclinical neuropharmacology, neurophysiology and existing clinical information, allowing testing of combinations in a virtual human patient [110]. Despite the tremendous therapeutic potential of multi-target drugs, their rational discovery and their development still represent a formidable challenge. In addition, translational barriers remain a problem either for single- or multi-target drugs, as discussed for AD in [111]. However, we foresee that new multi-target molecular entities, well characterized at the chemical, theoretical and biological level, offer a path to progress both in understanding causes of disease and in defining effective small molecule treatments.

## Abbreviations

AChE: acetylcholinesterase
AD: Alzheimer’s disease
ATC: Anatomical Therapeutic Chemical (Classification System)
Aβ: amyloid β
BACE-1: β-secretase1
BBB: blood–brain barrier
BuChE: butyrylcholinesterase
Cb: total brain concentration
Cu,b: unbound brain concentration
CDK: cyclin-dependent kinase
CNS: central nervous system
CYP: cytochrome P450
EGFR: epidermal growth factor receptor
FDA: Food and Drug Administration
FGFR: fibroblast growth factor receptors
FMPA: *N*-(furan-2-ylmethyl)-*N*-methylprop-2-yn-1-amine (a propargylamine inhibitor of MAO)
GPCR: G-protein coupled receptor
H3R: histone type 3 receptor
HBA: hydrogen bond acceptor
HBD: hydrogen bond donor
HCV: hepatitis C virus
HDAC: histone deacetylase
HER2: human epidermal growth factor receptor 2
5-HT: serotonin
5-HT4R: serotonin receptor type 4
LB-VS: ligand-based virtual screening
mAChR: muscarinic M1 acetylcholine receptor
MAO: monoamine oxidase
MoA: mechanism(s) of action
MTD: multi-target drugs
MTDL: multi-target designed ligand
NME: new molecular entities
NMR: nuclear magnetic resonance (NMR) spectroscopy
PD: Parkinson’s diseases
PDGFR: platelet-derived growth factor receptor beta
QSAR: quantitative structure–activity relationship
RET: mast/stem cell growth factor receptor kit (SCFR)
ROS: reactive oxygen species
SB-VS: structure-based virtual screening
SDAM: serotonin-dopamine activity modulator
VEGFR: vascular endothelial growth factor receptors
VS: virtual screening
